# New Approaches Based on Serial‐Block Face Electron Microscopy to Investigate the Peripheral Nervous System

**DOI:** 10.1111/jns.70019

**Published:** 2025-04-19

**Authors:** Vitalijs Borisovs, Mario Bossi, Laura Matino, Paola Marmiroli, Guido Cavaletti

**Affiliations:** ^1^ Experimental Neurology Unit School of Medicine and Surgery, Università di Milano‐Bicocca Monza Italy; ^2^ Faculty of Medicine Latvijas Universitāte Riga Latvia; ^3^ Istituto Degli Endotipi in Oncologia, Metabolismo e Immunologia “Gaetano Salvatore” (IEOMI) Consiglio Nazionale Delle Ricerche Naples Italy; ^4^ Fondazione IRCCS San Gerardo Dei Tintori Monza Italy

**Keywords:** dorsal root ganglia, peripheral nerve, scanning electron microscopy, serial block face

## Abstract

**Background and Aims:**

Serial block face scanning electron microscopy (SBF‐SEM) enables automated 3D imaging of specimens with ultrastructural resolution. However, its application is often restricted due to the complex and labor‐intensive nature of the processes involved. This study addresses the challenges associated with sample preparation and the final 3D reconstruction for ultrastructural analysis of peripheral nerves and dorsal root ganglia (DRG) specimens.

**Methods:**

Specimens from the caudal nerve and DRG of mice were prepared for SBF‐SEM using three different techniques: (1) manual high molecular weight staining, regarded as the gold standard, (2) automated standard transmission electron microscopy (TEM) preparation, and (3) automated uranyl‐free en bloc preparation. The acquired data were processed by combining different software programs for image analysis and 3D rendering.

**Results:**

Upon analyzing all samples, the high molecular weight method demonstrated its superiority. Nonetheless, the two alternative methods produced high‐quality images of the caudal nerve. Consequently, 3D rendering was successfully achieved for all samples using an automated approach. The investigation of DRG specimens posed greater challenges with the standard TEM preparation due to the low contrast of smaller organelles compared to the cytosol, whereas the uranyl‐free protocol provided significantly improved contrast.

**Interpretation:**

Our findings indicate that automated uranyl‐free staining can effectively compete with the traditional gold standard manual and uranyl‐based staining methods, albeit with some limitations. Furthermore, high‐quality SBF‐SEM imaging is attainable, especially in peripheral nerves, using samples prepared via the standard TEM method, thereby facilitating the analysis of previously embedded samples even if they were not specifically prepared for 3D examination.

## 1 Introduction

Recent advancements in imaging technologies have enabled the visualization of biological samples with unmatched precision. Transmission electron microscopy (TEM) has been a key tool in peripheral nervous system (PNS) research and diagnostics, allowing detailed descriptions of cell structure and organization due to its high resolution (down to a few nanometers) and high‐magnification images. While TEM can be used to reconstruct three‐dimensional structures by stacking ultrathin serial sections, the 2D nature of individual sections limits its ability to provide true volumetric information. Although 3D reconstructions from TEM data are possible, they require extensive manual alignment, making the process complex, time‐consuming, and less suitable for large‐scale sample analysis [1, 2, 3].

In this context, serial block face‐scanning electron microscopy (SBF‐SEM) offers an automated method for 3D specimen acquisition with ultrastructural resolution [4, 5]. This technique combines low‐voltage backscattered electron detection from layers beneath the sample surface with iterative slicing using an in‐chamber ultramicrotome, which cuts the sample in the z‐direction. As a result, 3D imaging provides a deeper and more accurate understanding of biological structures.

While SBF‐SEM has proven effective in central nervous system research, its use in the PNS remains limited. This is primarily due to the technique's labor‐intensive nature. Although SBF‐SEM enables faster volume imaging compared to other TEM‐based electron microscopy methods [6], sample preparation and data analysis pose significant challenges. En bloc staining, dehydration, and resin embedding require specific protocols that are largely manual, limiting throughput to a small number of specimens. Additionally, processing large data sets is computationally demanding and requires specialized user training [7].

Furthermore, since the staining and embedding methods used for SBF‐SEM are quite different from those for standard TEM preparation, the possibility of reusing PNS samples prepared for TEM in SBF‐SEM imaging has not been explored and remains uncertain.

Lastly, SBF‐SEM often employs uranyl acetate as a negative stain, which is radioactive and toxic, raising concerns about safety and environmental impact.

In this study, we tackle the challenges of sample preparation and 3D reconstruction by developing semi‐automated workflows for batch processing and volume rendering. We also investigate the feasibility of uranyl acetate‐free en bloc staining for ultrastructural analysis of peripheral nerves and dorsal root ganglia (DRG) specimens and the viability of using TEM‐prepared samples for SBF‐SEM imaging.

Specimens from the caudal nerve and DRG of mice were prepared for SBF‐SEM using three different techniques: (1) manual high molecular weight staining, regarded as the gold standard, (2) automated standard transmission electron microscopy (TEM) preparation, and (3) automated uranyl‐free en bloc preparation. The acquired data were processed by combining different software programs for image analysis and 3D rendering.

## 2 Materials and Methods

### 2.1 Tissues Processing

In compliance with the 3R's principles aimed at the reduction of animal use for experimental studies [8], healthy Balb/c mice (Envigo, San Pietro al Natisone, Italy) belonging as controls to a study conducted in conformity with the institutional guidelines in compliance with national (D. L.vo 26/2014, Gazzetta Ufficiale della Repubblica Italiana, n.61, March 14, 2014) and international laws and policies (European Union directive 2010/63/UE; Guide for the Care and Use of Laboratory Animals, U.S. National Research Council, 1996), and approved by the Italian Ministry of Health (approval n. 777/2022‐PR) were used. Intracardiac perfusion was performed in animals under deep anesthesia with Ketamine/Xylazine using a peristaltic pump (Heidolph Pumpdrive 5101, Biosigma, Cona, Venice, Italy). An 18‐gauge needle was inserted into the left ventricle at an angle approximately parallel to the midline of the heart; then a small incision was made in the right atrium. Immediately after the venous blood escaped from the right atrium (approx. 7 mL), perfusion with sterile saline solution was started at a constant speed of approximately 1 mL/5 s to clean the vascular tree. The animals were then perfused with 150 mL 4% paraformaldehyde at the same constant rate. Subsequently, caudal nerves and DRG were dissected out and immediately immersed in 3% glutaraldehyde diluted in 0.15 M sodium cacodylate buffer for 3 h for a further fixation. Nerves were cut into small pieces (approx. 5 mm) with a razor blade to help the samples fit inside the baskets of the automatic staining instrument and the staining solutions penetrate homogeneously within the tissue volume. DRG tissues were prepared without trimming their initial volume.

### 2.2 Manual and High Molecular Weight En Bloc Staining

The first set of samples was manually prepared and contrasted following the procedure described by Deerinck T.J. et al. [9] and adapted to our needs with modifications. Therefore, the fixative solution was replaced with 0.15 M sodium cacodylate buffer with three sequential 15 min washes. Samples were post‐fixed for 2 h in 2% osmium tetroxide/1.5% potassium ferricyanide K3[Fe(Cn)6], at room temperature with constant agitation. Thereafter, tissues were washed first in 0.15 M sodium cacodylate buffer and then with deionized water before leaving them in freshly filtered 1% thiocarbohydrazide aqueous solution for 40 min at 60°C. Specimens were further washed in water and impregnated with 1% osmium tetroxide in aqueous solution for 1 h at room temperature. Samples were subjected to additional contrast enhancement steps by soaking them overnight in a filtered solution of 1% uranyl acetate at 4°C, followed by Waltson's lead aspartate staining at 60°C for 30 min. Three 15‐min washes in water were performed and then followed by dehydration through graded ethanol balanced in deionized water (50%, 70%, 96%, two steps in 100%, 15 min each step), at room temperature. Two 10 min incubations in 100% propylene oxide were performed to ensure the total sample dehydration and were followed by embedding in epoxy resin at the hard formulation (13.75 mL of EMbed 812, 4.65 mL of Dodecenyl Succinic Anhydride (DDSA), 8.25 mL of Methyl‐5‐Norbornene‐2,3‐Dicarboxylic Anhydride (NMA), 533 μL of 2,4,6‐Tris(dimethylaminomethyl)phenol (DMP‐30)). Samples were placed into 50% propylene oxide/50% hard epoxy resin for 2 h and left overnight at room temperature with the open lid in a fume hood. The day after, the resin mixture was replaced with fresh and pure epoxy resin in hard formulation, devoid of any bubbles, for 2 h before transferring each sample into molds for the final resin polymerization at 60°C for 48 h, to create a block of uniform hardness.

### 2.3 Automated and Standard TEM Preparation

The second set of samples was processed with the Leica EM TP 1020 Automated Tissue Processor (Leica Biosystems, Buccinasco, Italy). All the samples were separately inserted into porous baskets, and reagent solutions were prepared and neatly placed for successive sample immersions. Solution penetration was helped by the gentle swirling of the baskets inside each reagent tank. The processing schedule was inspired by Palade G. et al. protocol [10] with modifications. After fixation, the fixative solution was replaced with phosphate buffer solution two times and, again, with 0.12 M sodium cacodylate buffer (pH 7.4) two times for 10 min each. Then, samples were impregnated with 1.5% osmium tetroxide diluted in the aforementioned buffer for 2 h and 30 min at room temperature. Specimens were consecutively washed in 0.12 M sodium cacodylate buffer for 10 min each prior to the gradual ethanol dehydration (50%, 70%, 96%, two steps in 100%, 10 min each step) at room temperature. Two incubations in 100% propylene oxide were performed for 10 min, followed by 66%, 33%, and 0% propylene oxide mixture balanced with epoxy resin for the gradual resin embedding (30 min each step) at room temperature. Resin was prepared by blending equal volumes of mixture A (9.4 g DDSA and 8.82 g EPON 812) and mixture B (8.14 g NMA and 12.5 g EPON 812), with 1.5%–2% of embedding medium accelerator (DMP‐30). Resin‐embedded specimens were finally transferred into molds, and the resin polymerized at 60°C for 48 h.

### 2.4 Uranyl Acetate‐Free En Bloc Staining With Automated Processor

The third set of samples was processed with the Leica EM TP 1020 Automated Tissue Processor (Leica Biosystems, Buccinasco, Italy). The processing schedule was inspired by Moscardini et al.'s published protocol [11] with modifications. The so‐called X‐solution (a lanthanide and phosphotungstic acid mixture) was produced in our laboratory following the description from the article. After specimen fixation was performed, samples were separately inserted into porous baskets with phosphate buffer and incubated for two times 10 min each. Successively, 0.12 M sodium cacodylate buffer (pH 7.4) was used to wash the samples for two times (10 min each) prior to post‐fixation in 1% potassium ferricyanide K3[Fe(Cn)6]‐reduced 1% osmium tetroxide for 1 h and 45 min at room temperature. Specimens were then washed twice in 0.12 M sodium cacodylate buffer and again in bidistilled water (each step for 10 min). Samples were then conditioned in 20% ethanol for 10 min before being impregnated with X‐solution for 1 h at room temperature. Gradual ethanol dehydration (20%, 50%, 70%, 96%, two steps in 100%, 10 min each step) was then performed at room temperature. Samples were subsequently impregnated in 100% propylene oxide for 10 min and gradually embedded in epoxy resin with propylene oxide and resin balanced mixture (75%, 50%, 25%, and 0%, respectively) for 30 min each step, at room temperature. The used resin had a hard formulation. Resin‐embedded specimens were finally transferred into molds, and the resin polymerized at 60°C for 48 h.

### 2.5 From Block to Sample Mounting

Semi‐thin (1–1.5 μm) sections from the block face were collected, stained with toluidine blue, and examined under a conventional bright field optical microscope for structural integrity check and selection of the region of interest (ROI); ultrathin (approx. 70 nm) sections were checked using a Hitachi TEM HT7800 (Hitachi High‐Tech Europe GmbH, Krefeld, Germany) before proceeding to sample mounting and SBF‐SEM imaging, to ensure the quality of the staining. The block face was, hereafter, trimmed with a razor blade to create a truncated square pyramid (approx. 500 μm × 500 μm × 2 mm) with the ROI near its center and to remove the empty resin which increases the build‐up of negative charges on the surface during the imaging. The pyramid was then dislodged and mounted onto an aluminum pin using a drop of pre‐cured embedding resin as glue. The resin drop was left polymerizing overnight in the oven at 60°C to ensure sample‐pin anchoring. The specimen surface was again smoothed with the diamond knife, to produce a flat block face and reduce any possible small tilts with the diamond knife of the in‐chamber ultramicrotome. Finally, mounted samples were sputter‐coated with a fine nm‐layer of gold palladium using a Vac Coat DSCT sputter coater device (Vac Coat Ltd., London, UK) and the aluminum pin base and block edges were covered with a small amount of silver paste before the insertion into the SBF‐chamber.

### 2.6 High Resolution 3D Imaging via SBF‐SEM

Cubic microns of imaging data were achieved with a Zeiss GeminiSEM 360 (Carl Zeiss S.p.A., Milan, Italy) equipped with the in‐chamber ultramicrotome Volutome, and piloted by the Volutome software package, which fully assists the slicing and imaging cycle with minimal user involvement. Sectioning was fixed to the nominal speed of 0.1 mm/s and thickness of 50 nm for every cycle, thus determining the axial resolution as well as the voxel size. In parallel, high‐resolution acquisitions of resin block faces were performed using a 30 μm beam aperture and the Volutome backscattered electrons (BSD) detector, which allows image acquisition with hundreds‐μm field‐of‐view. In particular, for DRG acquisition, the pixel size was always chosen above 10 k per side for a larger sample surface vision. Optimum imaging conditions were adjusted depending on the sample level of contrast, conductivity, and possible sample damage due to negative charging. Nitrogen gas injection through the focal charge compensation (FCC) system was systematically used to mitigate charging and improve cutting performance.

### 2.7 Image Analysis and 3D Rendering

Acquired data were processed by combining different software programs. If needed, image stack alignment was performed using the Zen Blue software (Carl Zeiss S.p.A., Milan, Italy); segmentation, deep learning models, final 3D rendering, and downstream analysis of tissue ultrastructure were, instead, accomplished via Zen Arivis Vision4D (Carl Zeiss S.p.A., Milan, Italy). The fully automated approach was chosen to post‐process different sample acquisitions. Therefore, for each tissue compartment (i.e., myelin sheaths, axons, mitochondria, cell nucleus, …), deep learning (DL) models were devised by manually tracing the structures of interest with the brush tool; generally, up to 50 annotations were needed for data segmentation. Regardless of the used staining strategy, the single biological compartment was identified and reconstructed with the same model. DL segmenters were then combined with an additional feature filter to remove erroneously labeled objects prior to the 3D reconstruction.

### 2.8 Contrast Evaluation

Differences in contrast between cell organelles and surrounding cytoplasm were evaluated using Fiji software (Open‐source ImageJ distribution. Available at www.imagej.net). Pixel gray values along a line (1‐pixel width and approx. 0.5–0.7 μm length) of three elements from five different SEM frames (*n* = 15) crossing an organelle and adjacent cytoplasm region were plotted with Graph Pad software v.10.0.2 (GraphPad, La Jolla, CA). Maximum values correspond to less contrasted regions, such as the cytosol; on the contrary, minimum values correspond to higher heavy metal deposition, as in the case of lipidic membranes.

## 3 Results

### 3.1 Caudal Nerve Examination

**FIGURE 1:**
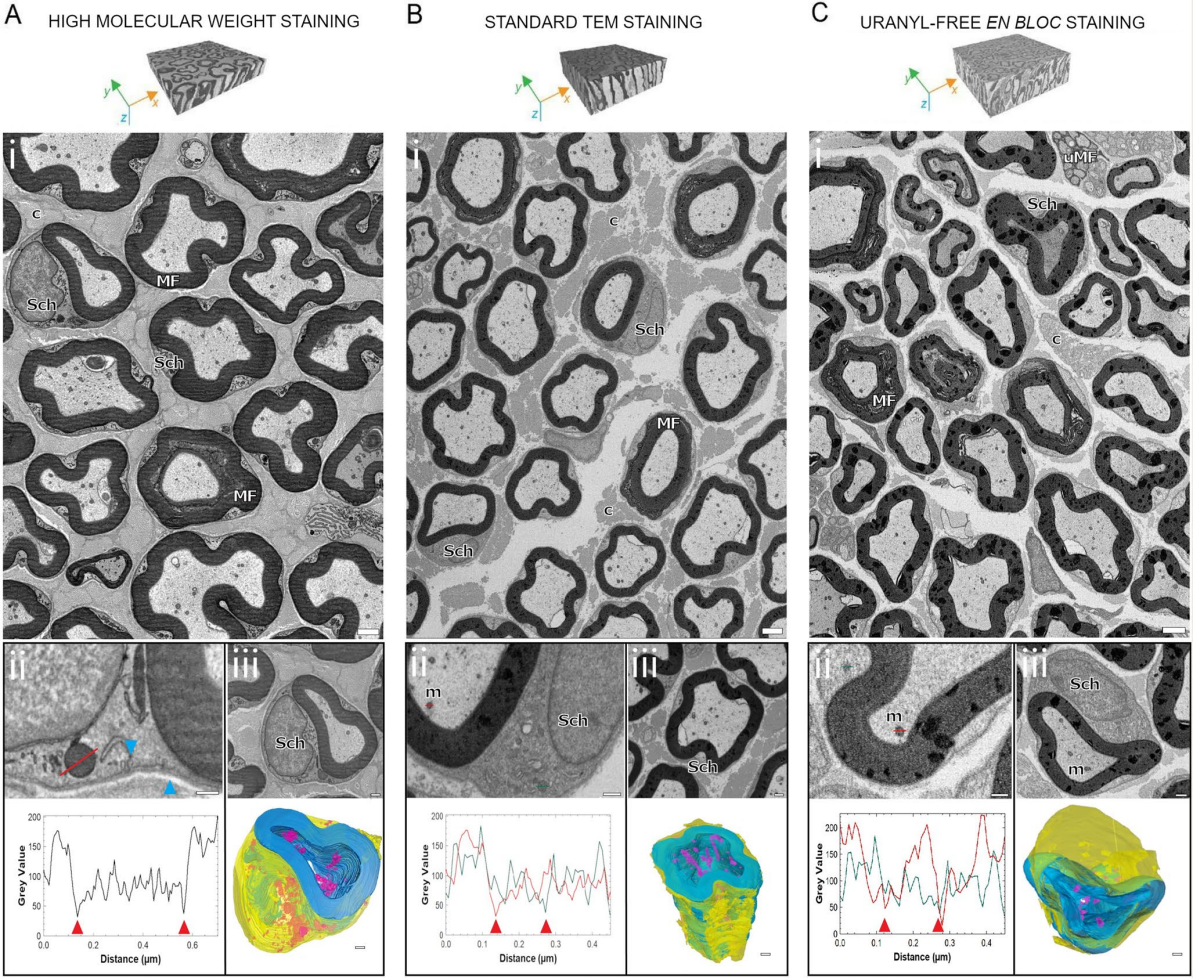
Caudal nerve. Distinctive biological structures are highlighted: Myelinated fibers (MF), unmyelinated fibers (uMF), intra axonal mitochondrion (m), collagen bundles (c), and Schwann's cells (Sch). (A) high molecular weight staining: (i) micrograph from dataset acquired in BSD mode with 3 kV as accelerating voltage, 5 nm as x, y resolution, 1.6 μs as dwell time, 60% FCC and resulting electron dose approx. 94 e/nm^2^. Scale bar 2 μm. (ii) Magnified view of Schwann's cells organelles and of a large endocytic vesicle close the plasma membrane (pointed by blue arrow, scale bar 500 nm). Difference in contrast (grey values profile) between the Schwann cells vesicle and cytosol is plotted and measured along a 1‐pixel line crossing the organelle. Maximum values correspond to cytosol, minimum values pointed by red arrows correspond to organelle membranes. (iii) View of a myelinated axon and of a Schwann cell (scale bar 1 μm) with the 3D reconstruction (volume size of 10 × 9 × 20 μm) of myelin (in blue), Schwann cell (in yellow) and mitochondria (in magenta). Scale bar 1 μm. (B) Standard TEM staining: (i) micrograph of a caudal nerve (BSD mode, 4 kV, 6 nm pixel size, 2 μs as dwell time, 100% FCC, approx. 84 e/nm^2^). Scale bar 2 μm. (ii) Magnified view of a Schwann cell and intra‐axonal mitochondria (scale bar 1 μm) with the plot of their grey values (red line for intra‐axonal mitochondrion, green line for Schwann's cell mitochondrion). (iii) View of myelinated axon and nearby Schwann cell (scale bar 1 μm), with the 3D reconstruction of myelin (in blue), Schwann cell (in yellow) and mitochondria (in magenta) (volume size of 12 × 11 × 20 μm, scale bar 1 μm). (C) Uranyl‐free en bloc staining: (i) micrograph of a caudal nerve (BSD mode, 1.75 kV, 6 nm pixel size, 1 μs as dwell time, 65% FCC, approx. 40 e/nm^2^). Scale bar 2 μm. (ii) Magnified view of a Schwann cell and intra‐axonal mitochondria (scale bar 1 μm) with the plot of their grey profiles (red line for intra‐axonal mitochondrion, green line for SC's mitochondrion). (iii) Magnified view of myelinated axon and nearby Schwann cell (scale bar 500 nm), with the 3D reconstruction of myelin (in blue), Schwann cell (in yellow) and mitochondria (in magenta) (volume size of 24 × 24 × 20 μm, scale bar 1 μm).

The en bloc staining method described by Deerink et al. [9], used in preparing the first set of samples, has been reported to provide exceptional structural detail in biological specimens [12]. As a result, we considered it the gold standard for our SBF‐SEM imaging. Figure 1Ai presents a micrograph of the caudal nerve's inner architecture, prepared using this high molecular weight staining technique with slight modifications. The heavy metal deposition enhanced the resolution of cellular ultrastructure, intra‐axonal mitochondria, and even individual collagen filaments, achieving a minimum x, y pixel size of 4–5 nm. Figure 1Aii provides a magnified view of a Schwann cell segment, clearly showing the double‐layered nuclear membrane, vesicles, and intracellular organelles. A plot of pixel intensity variations along a cross‐sectional line demonstrates distinct intensity differences, where cytosolic regions exhibit the highest values and organelle bodies the lowest. The conductivity achieved through this sample preparation enabled long‐duration imaging with low‐voltage stimulation (2.5 keV) while minimizing charge accumulation, which was controlled using FCC at a medium power level (~60%). Figure 1Aiii shows a myelinated axon and the adjacent Schwann cell, demonstrating optimal visualization of intracellular organelles and membranes.

In addition to manual heavy metal staining, we explored the use of automated sample preparation methods in the second set of samples.

First, we adapted the Palade et al. [10] protocol to suit our needs. While these samples exhibited only moderate heavy metal impregnation, various tissue structures remained clearly distinguishable under low magnification, as illustrated in Figure 1Bi. Myelin sheaths displayed the strongest contrast with a uniform intensity. Similarly, mitochondria within axons were easily identifiable due to their distinct contrast relative to surrounding structures, while Schwann cell nuclei, unmyelinated axons, and endoneural collagen bundles were visible with a fixed x, y pixel size of 6 nm—our lowest achievable lateral resolution using this staining method. At higher magnifications, intracellular structures in Schwann cells were generally stained, though individual organelles remained difficult to resolve due to the limited contrast. As shown in magnified Figure 1Bii, the contrast difference between the cytosol and organelles was minimal (green line), whereas mitochondria within axons exhibited a stronger distinction (red line). Imaging with an accelerating voltage below 4 keV provided little enhancement in structural detection, so the electron dose (approx. 84 e/nm^2^) was minimized using FCC at maximum power.

For the third set of samples, we employed an uranyl acetate‐free staining method. This staining approach, known as X‐solution, was developed by Moscardini et al. [11] and consists of a lanthanide and phosphotungstic acid mixture, which enhances electron beam scattering. Myelin, axonal mitochondria, and Schwann cells, as well as nuclei from other cells within the nerve, were identifiable at a maximum resolution of 5–6 nm (Figure 1Ci and with higher magnification and intensity profiles in Figure 1Cii), even at a reduced electron beam stimulation of 1.75 keV. However, other intracellular structures exhibited lower contrast, making them harder to differentiate from the cytosol, as seen in Figure 1Cii.

We trained separate DL models in the Arivis Vision4D Pro environment to segment myelin, Schwann cells, and mitochondria across all three staining methods (3D renderings shown in Figure 1Aiii,Biii,Ciii; see also Video S1). Myelin structures, which strongly retain osmium, were efficiently segmented due to their relatively consistent shape across sections (Video S2). While Schwann cells displayed variable wrapping morphologies along myelin and shared contrast similarities with collagen bundles, their segmentation remained accurate thanks to well‐defined membrane boundaries. Mitochondria segmentation was highly effective when they were within axons, where the contrast difference with surrounding structures was pronounced (Figure 1Aiii,Biii,Ciii). However, recognition was less reliable for mitochondria located within the cytoplasm of cell bodies, where the contrast was weaker. This led to increased uncertainty in the deep‐learning algorithm, requiring more extensive quality control, particularly for smaller mitochondria.

### 3.2 DRG Examination

**FIGURE 2:**
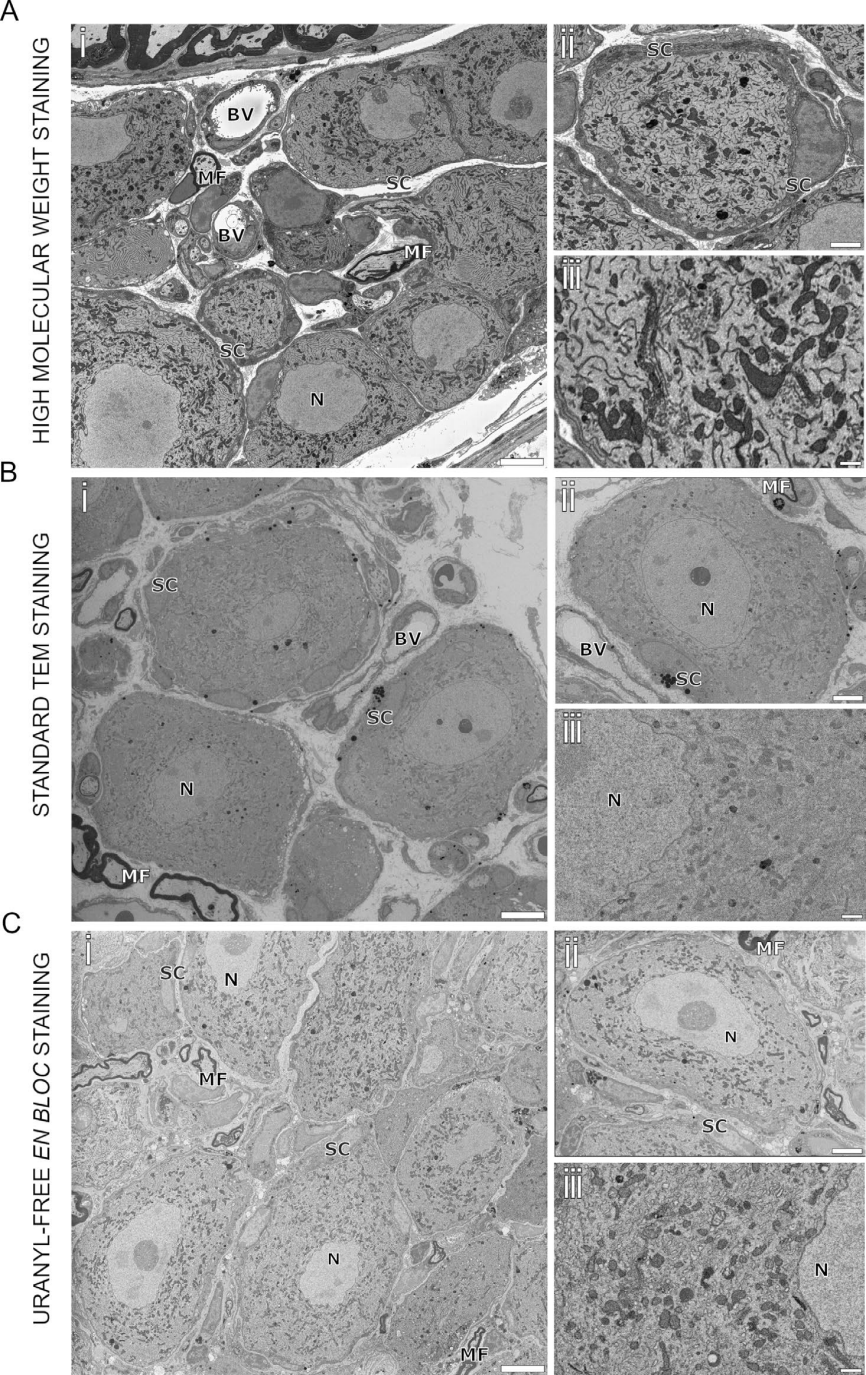
Dorsal root ganglia. Distinctive biological structures are highlighted: Neuronal nucleus (N), blood vessels (BV), myelinated fibers (MF), and satellite cells (SC). (A) high molecular weight staining: (i) DRG samples acquired in BSD mode with 2.5 kV as accelerating voltage, 5 nm as x, y resolution, 1.2 μs as dwell time, 100% FCC and resulting electron dose approx. 69 e/nm^2^. Scale bar 10 μm. (ii) Contrast difference between neuron and satellite cell allow clear distinction between the two cells. Bar 5 μm. (iii) Magnified view of DRG neuron ultrastructure. Scale Bar 2 μm. (B) Standard TEM staining: (i) DRG samples acquired in BSD mode with 3.15 kV as accelerating voltage, 7 nm as x, y resolution, 1.6 μs as dwell time, 100% FCC and resulting electron dose approx. 48 e/nm^2^. Scale bar 10 μm. (ii) Entire view of a neuron enveloped by a satellite cell with minimal contrast difference. Scale bar 5 μm. (iii) Magnified view of DRG neuron ultrastructure. Scale bar 2 μm. (C) Uranyl‐free en bloc staining: (i) DRG samples acquired in BSD mode with 2 kV as accelerating voltage, 6 nm as x, y resolution, 1 μs as dwell time, 100% FCC and resulting electron dose approx. 39.6 e/nm^2^. Scale bar 10 μm. (ii) Entire view of a neuron enveloped by a satellite cell. Scale bar 5 μm. (iii) Detailed DRG neuron ultrastructure is clearly visible. Scale bar 2 μm.

The first set of samples, prepared with heavy metal deposition, provided excellent contrast and fine ultrastructural details that were clearly revealed, with key features such as nuclear envelopes, mitochondria, and the endoplasmic reticulum easily distinguishable from the cytoplasm (Figure 2Ai). Cell membranes remained sharp even after more than 200 sectioning steps (Figure 2Aii). These results were consistently achieved using a low accelerating voltage of 2.5 kV and medium FCC settings. The combination of heavy metals was particularly effective in distinguishing closely packed cell types, such as satellite glial cells and neurons (Figure 2Aiii), with a fixed pixel size of 5 nm. However, while the staining method provided exceptional detail in individual slices, the densely packed and complex structure of DRG tissue made achieving smooth 3D reconstructions more challenging than in the caudal nerve. Overlapping structures and inconsistent contrast along the z‐axis presented significant obstacles in generating accurate volumetric models.

In contrast, the second set of samples, prepared using standard TEM staining, exhibited reduced contrast and structural clarity. Imaging was conducted with an accelerating voltage of up to 3.15 kV, achieving a resolution of 6 nm per pixel. While larger structures such as nuclei, nucleoli, and myelin were visible, finer details—including organelles and the boundaries between satellite and neuronal cells—were often blurred due to minimal gray‐level differences (Figure 2Bi–iii). The low contrast between adjacent structures made manual annotation difficult and posed an even greater challenge for automated neural networks. These limitations were particularly evident in regions with densely packed cells, where distinguishing individual features and 3D reconstruction became increasingly complex.

The third set of samples, stained en bloc with X‐solution, produced significantly sharper and more detailed images. The enhanced scattering properties of this staining method enabled imaging at very low voltages (1.5–2 kV) while maintaining a spatial resolution of 5–6 nm. Using FCC at maximum power further reduced charging artifacts, resulting in highly detailed views of organelles, nucleoli, myelin, and entire cell bodies across both the sample surface and deeper layers (Figure 2Ci–iii). While these improvements facilitated individual structural analysis, reconstructing 3D models remained challenging. The dense and heterogeneous composition of DRG tissue made it difficult to achieve consistent and reliable volumetric reconstructions.

## 4 Discussion

Accurate sample preparation is crucial for achieving high‐quality ultrastructural analysis, as many cellular structures remain difficult to visualize due to their inherently low contrast [13]. Additionally, proper preparation is essential for supporting sectioning [14]. The specific requirements of SBF‐SEM add another layer of complexity, often making the process labor‐intensive and time‐consuming. Standard SBF‐SEM protocols typically rely on uranyl‐based staining, but replacing this with an effective, non‐radioactive alternative would improve safety in sample preparation for ultrastructural imaging.

In this study, we compared the ultrastructural contrast achieved with the gold‐standard manual uranyl‐based staining method from Deerink et al. [9] to two alternative protocols using an automated tissue processor. This system enables the preparation of hundreds of samples through precise, time‐controlled impregnation steps. The first alternative used a standard TEM preparation to assess whether previously prepared samples could be analyzed with SBF‐SEM, despite not being specifically designed for this technique. The second tested the feasibility of a uranyl‐free staining approach.

To evaluate the effectiveness of these methods in studying the PNS, we analyzed both the caudal nerve and DRG. These sample types were selected due to their distinct histological structures, allowing for a broader comparison in imaging and 3D reconstruction. Notably, DRG samples exhibited significant charging effects under SBF‐SEM conditions, primarily due to their relatively low lipid content compared to the highly myelinated caudal nerve. Therefore, we also assessed the effectiveness of FCC in mitigating this technical challenge.

Our results demonstrated that automated tissue processing ensures consistent contrast enhancement within each sample and provides reproducible staining across batches. The time required for specimen preparation was significantly reduced to just one day—plus the time needed for resin polymerization—compared to the four full days required by manual methods. User involvement was limited to reagent preparation, sample loading, and unloading. Additionally, automation minimized reagent consumption, as multiple specimens could be processed simultaneously in shared solution tanks.

When compared to the gold standard [9], which remains superior in resolving ultrastructural details of various compartments and organelles, the standard TEM preparation and the uranyl‐free en bloc method yielded reliable results for caudal nerve samples—particularly when examining structures larger than 50 nm with high contrast relative to the background. However, these two alternative methods, especially the standard TEM preparation, were less effective for DRG analysis.

The electron doses we applied exceeded values recommended by the manufacturer and previous studies [15, 16], leading to improved image quality in low‐contrast specimens [15, 17]. We identified an optimal balance between minimizing charging effects and achieving high‐resolution imaging for both moderately charge‐prone (caudal nerve) and highly charge‐prone (DRG) samples. Notably, less conductive regions—such as cell nuclei, blood vessel lumens, and occasional bare resin areas—rarely exhibited charge‐related artifacts, unlike previous observations [12, 14]. FCC played a critical role in this outcome, enhancing imaging quality and increasing the versatility of SBF‐SEM, even for challenging specimens.

For all sample types, we observed that negative charge accumulation decreased after a few days in the SEM chamber. This allowed us to acquire data at smaller x and y pixel sizes within that timeframe. Additionally, samples prepared using the first and third protocols incorporated hard resin formulations, which are known to better support serial sectioning [5, 12]. However, no significant surface alterations were detected on specimen block faces, regardless of whether soft or hard resin was used, as confirmed by secondary electron imaging (data not shown). This stability was likely due to the combination of FCC discharge control [18] and oscillating knife cutting mode [19].

Our data processing pipeline streamlined post‐processing, improving workflow efficiency for image analysis and 3D rendering. For each tissue type, we developed a deep learning (DL) model to reconstruct specific biological structures, ensuring adaptability to variations in imaging parameters and staining conditions. Each model required approximately 50 annotations to achieve accurate segmentation, with occasional refinements necessary to enhance quality. Results were promising, particularly for well‐defined structures such as myelin and Schwann cells, which benefited from strong osmium staining. However, segmentation proved more challenging for smaller or delicate structures, especially in samples processed using the standard TEM preparation, where contrast was lower.

In conclusion, we demonstrated that automated uranyl‐free staining combined with hard resin embedding can serve as a viable alternative to the gold‐standard manual uranyl‐based staining technique, albeit with some limitations. Furthermore, we confirmed that good‐quality SBF‐SEM imaging is possible with peripheral nerve samples prepared using standard TEM protocols, enabling the analysis of previously embedded specimens even if they were not specifically prepared for 3D imaging.

## Supporting information


**Video S1.** Uranyl‐free stained sample (X‐sol). Reconstruction example of two full axons with blue—myelin, yellow—Schwann’s cells, magenta—mitochondria. Surface quality set to native with the transparency setting on. Moscardini et al. protocol [11], FullHD (1920 × 1080).

**Video S2.** Uranyl‐free stained sample (X‐sol). Reconstruction of a single axonal myelin (blue). Native surface quality without transparency setting. Moscardini et al. protocol [11], FullHD (1920 × 1080).

## Data Availability

The data that support the findings of this study are available from the corresponding author upon reasonable request.
